# Conceptualising a Community-Based Response to Loneliness: The Representational Anchoring of Nature-Based Social Prescription by Professionals in Marseille, Insights from the RECETAS Project

**DOI:** 10.3390/ijerph22091400

**Published:** 2025-09-07

**Authors:** Lucie Cattaneo, Alexandre Daguzan, Gabriela García Vélez, Stéphanie Gentile

**Affiliations:** 1 Service d’Evaluation Médicale, Hôpital sainte Marguerite, Assistance Publique des Hôpitaux de Marseille, 13009 Marseille, France; lucie.cattaneo@ap-hm.fr (L.C.); alexandre.daguzan@ap-hm.fr (A.D.); 2 City Preservation Management Research Group, Faculty of Architecture and Urbanism, University of Cuenca, Cuenca 010150, Ecuador; gabriela.garcia@ucuenca.edu.ec; 3 School of Medicine, Aix Marseille Université, SSA, RITME, 13005 Marseille, France

**Keywords:** loneliness, social isolation, nature-based social prescription, social representations, qualitative research, urban inequalities, participatory approaches, health promotion, health inequalities

## Abstract

Background: Urban loneliness is rising worldwide and is a recognised public-health threat. Nature-Based Social Prescriptions (NBSPs), guided group activities in natural settings, are being piloted in six cities through the EU project RECETAS. However, in new contexts such as Marseille, its implementation is constrained by professionals’ limited knowledge of the concept. Objectives: (i) Exploring how professionals in Marseille (France) conceptualise NBSPs; (ii) Identifying perceived facilitators and barriers to implementing NBSPs among residents facing social isolation and loneliness. Methods: Twelve semi-structured interviews were conducted with health, social-care, and urban–environment professionals selected via network mapping and snowball sampling. Verbatim transcripts underwent inductive thematic analysis informed by Social Representation Theory, with double coding to enhance reliability. Results: Five analytic themes emerged: (1) a holistic health paradigm linking nature, community, and well-being; (2) stark ecological inequities with limited green-space access in deprived districts; (3) work challenges due to the urgent needs of individuals facing significant socio-economic challenges in demanding contexts; (4) a key tension between a perceived top-down process and a preference for participatory approaches; (5) drivers and obstacles: strong professional endorsement of NBSPs meets significant systemic and institutional constraints. Conclusions: Professionals endorse NBSPs as a promising approach against loneliness, provided programmes tackle structural inequities and adopt participatory governance. Results inform the Marseille RECETAS pilot and contribute to global discussions on environmentally anchored health promotion.

## 1. Introduction

Loneliness, defined as the perceived gap between desired and actual social relationships, has become a major public-health determinant [1]. In France, the proportion of adults who report feeling lonely “often or almost every day” rose from 19% in 2020 to 29% in 2022 [2]. This rise mirrors global trends driven by rapid urbanisation, fragmentation of family ties and, more recently, COVID-19 distancing measures. Chronic loneliness is linked to a 26% increase in all-cause mortality and is an established risk factor for cardiovascular disease, depression, and cognitive decline [3]. Despite substantial evidence, loneliness remains insufficiently acknowledged in public health and social policy and continues to be predominantly conceptualised as an individual deficit rather than as the outcome of structural social, economic, or environmental determinants [2,4,5,6].

Addressing loneliness, therefore, requires systemic, cross-sectoral interventions that tackle its social and environmental determinants. Social prescribing, as developed within the UK National Health Service, connects individuals to non-medical community activities, such as creative workshops, walking groups or volunteering, and has shown positive effects on well-being and social connectedness [7,8]. Among the different forms of social prescribing, Nature-Based Social Prescription (NBSP) offers unique potential by leveraging the restorative, socially connective, and health-promoting qualities of natural environments. Rooted in the framework of the social determinants of health (SDH), it seeks to address the combined effects of social isolation, socio-economic deprivation, limited access to healthcare, and territorial fragmentation in urban settings [9].

In Marseille, France’s second-largest city, these challenges are concentrated in the northern districts, where most of the 38 Priority Neighbourhoods (Quartiers Prioritaires de la Ville, QPV) are located and poverty rates are more than double the national average [10,11]. These areas, shaped by successive migration waves, are home predominantly to residents of North African, sub-Saharan African and Comorian origin [12]. They experience intersecting forms of disadvantage, including economic hardship, territorial stigmatisation, language barriers and limited administrative literacy, which restrict social support networks and reinforce structural isolation [13]. Vulnerability is not evenly distributed: young people, low-income older adults, people with disabilities or chronic illness, and single-parent families in precarious situations are particularly overrepresented among socially isolated residents [14].

Mobility and environmental inequalities further compound these challenges. Although Marseille is five times larger than Lyon, its public transport network extends only 30 km compared to 73 km in Lyon. In the most deprived districts, 40% of households lack a private vehicle, limiting access to employment, services and social participation [15]. Green space provision is equally unequal: despite the Calanques National Park covering 20% of the city, Marseille offers only 5 m^2^ per inhabitant, six times less than Lyon and far below the WHO minimum recommendation of 9 m^2^ [16,17]. For many vulnerable residents in northern districts, these combined mobility and environmental constraints make nature both physically and symbolically distant, despite its well-documented benefits for health and social connection.

In this context, loneliness in Marseille reflects the combined impact of social, territorial, and environmental inequalities. This intersection is precisely where the European project RECETAS (Reimagining Environments for Connection and Engagement: Testing Actions for Social Prescribing in Natural Spaces) operates. Launched in 2021, RECETAS pilots NBSP interventions in six cities: Barcelona, Cuenca, Helsinki, Marseille, Melbourne, and Prague. The project’s central aim is to reduce loneliness in urban settings by testing and evaluating an innovative intervention based on NBSP [18]. In Marseille, the focus is on residents of deprived neighbourhoods who face migration-related barriers and multiple, overlapping forms of exclusion, placing them at heightened risk of chronic loneliness [19]. To adapt the intervention to this local context, RECETAS designed a co-creative process with local actors, structured in the following three progressive phases: diagnosis, participatory diagnosis, and co-creation [19]. The initial diagnosis phase sought to build a foundational understanding of the local context, its challenges, and the network of actors able to propose nature-based solutions, before the intervention design was co-developed with beneficiaries in later phases.

As part of this initial diagnosis phase, NBSP was found to be largely unfamiliar: a 2021 mapping of local actors showed that 89% of professionals in Marseille did not know the term. Understanding how such an unfamiliar concept is interpreted requires an analytical framework that explains how new ideas are made meaningful. Social Representation Theory (SRT) provides such a framework, with the concept of anchoring describing how novel interventions are incorporated into pre-existing knowledge, values, and practices [20,21]. By situating the study within SRT, the analysis not only explores local perceptions of NBSPs but also aims to illuminate processes and dynamics that may inform its adoption in other urban contexts facing comparable socio-environmental challenges. The objective of this study is, therefore, to explore how professionals in health, social care, and urban–environment sectors conceptualise NBSP at the outset, and to identify perceived facilitators and barriers to its implementation.

## 2. Methods

### 2.1. Study Design

This study adopted an exploratory qualitative approach. The methodology was grounded in a constructivist and interpretivist epistemological stance, which holds that knowledge is co-constructed through interaction and shaped by social and professional contexts. In this perspective, reality is not considered fixed and objective, but rather the product of shared meanings emerging through dialogue between participants and researchers. The constructivist stance emphasises that these meanings are context-dependent, while the interpretivist stance focuses on understanding the subjective experiences and interpretations of individuals within their specific socio-professional environments.

This positioning aligns with the theoretical framework of Social Representation Theory, particularly the process of anchoring, through which a new social object, such as NBSP, is made meaningful based on pre-existing knowledge and shared understandings held by individuals, for instance, regarding conceptions of health and nature [22]. Based on the principle that this knowledge is mobilised by actors and accessible through individual discourse, an inductive approach using research interviews made it possible to explore the categories and meanings used to construct the concept of NBSPs [23]. Accordingly, the research design privileged open-ended, flexible questioning and reflexive analysis to capture the diversity of perspectives. This approach also allowed for the co-construction of meaning throughout the data collection process. The holistic nature of qualitative inquiry is especially well-suited to capturing the complexity involved in integrating an emerging concept such as NBSP across different professional domains, where disciplinary boundaries, limited institutional recognition, and unfamiliarity with the approach may challenge its implementation. The study is reported in accordance with the COREQ (Consolidated Criteria for Reporting Qualitative Research) checklist [24].

### 2.2. Participants and Recruitment

Participant recruitment was based on a 2021 mapping of local actors. This mapping took the form of a Social Network Analysis (SNA), conducted in collaboration with Visible Network Labs via the PARTNER platform. Its objective was to understand how organisations in the pilot cities addressed loneliness and well-being through social prescribing and nature-based activities. For the Marseille context, this analysis identified a network of 392 organisations, from which the 12 professionals were ultimately selected to participate in this study based on the inclusion criteria (see Table 1).

Drawing on the Marseille dataset, the sample was constructed using purposive sampling, supplemented by snowball sampling [25], where initial participants meeting the inclusion criteria were first identified from the SNA list and invited to participate. This extended recruitment to actors not captured in the original mapping and ensured sectoral diversity across fields such as health, social work, urban planning, and environmental NGOs. The inclusion criteria were twofold: (1) representing key NBSP domains (health, social action, urban nature) and (2) holding either a decision-making or operational role, to capture representations across different hierarchical levels. Recruitment and preliminary analysis were conducted iteratively, with interviews transcribed and coded in parallel to identify emerging themes. Thematic saturation was considered reached when two consecutive interviews yielded no new codes or substantial insights relevant to the research objectives. This point occurred after the tenth interview, with two additional interviews conducted to confirm it. This resulted in a total of twelve participants [26].

### 2.3. Data Collection

Twelve semi-structured interviews (SSIs) were conducted in person, each lasting between 45 and 75 min. The interviews took place at participants’ workplaces or, in two cases, in neutral public spaces to ensure confidentiality and participant comfort. All professionals were informed of the study’s objectives and the voluntary nature of their participation. They were also informed that no nominative data (direct or indirect) would be collected. Verbal informed consent was obtained prior to each interview, in accordance with ethical standards for minimal-risk qualitative research [27]. The NBSP intervention in Marseille was approved by the Ethics Committee of the University of Aix-Marseille (N/Ref: 2023-01-05-03). With participants’ authorisation, all interviews were audio-recorded and transcribed verbatim for analysis. The interviews were conducted by a health psychologist trained in qualitative methods and discourse analysis, ensuring both methodological consistency and sensitivity to the personal and professional dimensions explored. The interviewer maintained a reflexive stance throughout the research process, acknowledging her professional background in health psychology and prior involvement in the RECETAS project, and considering how these might influence data collection and interpretation. The wider research team, which included public health specialists, social scientists, and environmental health experts, regularly discussed how their diverse disciplinary backgrounds, professional experiences, and positions within the project could shape the interpretation and emphasis of specific themes. A reflexive journal was kept to document potential biases and analytical decisions. Each interview began with a free-association task in which professionals were asked to spontaneously cite four words or ideas that came to mind upon hearing the term “Nature-Based Social Prescription”. (prompt: “Please tell me the four words, expressions, or ideas that spontaneously come to mind when you hear ‘Nature-Based Social Prescription’”). This technique, commonly used in research on social representations, is designed to elicit immediate associations and latent representations prior to engaging in a guided discussion [23]. Unlike some approaches that analyse these evocations in isolation, it was decided to incorporate them directly into the broader thematic analysis, consistent with a holistic interpretation of meaning construction on a new social object such as NBSP.

The remainder of the interview followed a semi-structured guide organised around five thematic areas: (1) professional role and positioning; (2) professional experiences with the target populations; (3) perceptions of loneliness and well-being; (4) the role of nature in health and social action; (5) conditions for the appropriation of NBSP and the involvement of target groups. The guide was developed collaboratively by the research team, piloted with two professionals from outside the study sample, and refined for clarity and relevance before data collection began.

### 2.4. Data Analysis

All interviews were transcribed verbatim in their original language (French) and analysed using an inductive thematic analysis following a six-step approach [28]. Data management and coding were facilitated using NVivo 12 software (QSR International, Melbourne, Australia), which allowed for the systematic organisation of codes and themes. After an initial immersion phase involving repeated readings of the transcripts, each interview was systematically coded into distinct units of meaning, which were then grouped to form potential initial themes. To ensure reliability, this initial coding was conducted independently by two health psychologists, who then met to compare their coding, resolve discrepancies, and establish a consensual coding scheme. This scheme was subsequently applied to the entire dataset, focusing on identifying commonalities and differences across interviews. A comparative matrix in NVivo mapped convergences and divergences in coding, allowing the naming and hierarchical organisation of final themes and sub-themes. The process also examined differences and similarities in representations according to professional sectors (health, social, urban/environment) and hierarchical roles (operational vs. decision-making).

This combined integration of spontaneous discourse from the free-association task and more developed reflections enabled a nuanced interpretation of the anchoring processes shaping professionals’ emerging representations of NBSP. As an additional validation strategy, preliminary themes were discussed within the research team to ensure interpretive coherence and minimise individual bias. However, no formal member checking with participants was conducted, due to time constraints and the exploratory nature of the study, which prioritised timely thematic synthesis over iterative participant validation.

## 3. Results

Thematic content analysis revealed five major themes and thirteen sub-themes, as detailed in Table 2. These themes are: (I) a holistic conception of health, community, and nature; (II) structural inequalities in Marseille; (III) populations facing cumulative disadvantage; (IV) pathways toward participation and recognition; (V) barriers and facilitators to NBSP implementation.

### 3.1. A Holistic Conception of Health, Community, and Nature

This first theme comprises three sub-themes: an ecosystemic view of health, the fundamental role of social belonging, and nature as a vital and socially mediating entity, each with distinct yet interconnected dimensions.

Professionals consensually articulated an integrated vision of health, nature, and community. Health was unanimously described as a dynamic ecosystem involving multiple interacting factors. One professional explained that basic needs like eating and housing are often considered priorities, “without realizing that this is already part of global health” (SSI 9). In this ecosystemic view of health, the focus was on the interdependence between physical, social, and environmental conditions as structural determinants of well-being, rather than on isolated factors. This perspective primarily emphasised the systemic and environmental context in which health is maintained.

Following this, the social belonging dimension, raised by 11 out of 12 professionals across all sectors, referred specifically to the perceived need to feel part of a group or community. Whereas the ecosystemic view addresses macro-level interconnections between health, society, and the environment, social belonging was described at the micro- and meso-levels, focusing on interpersonal bonds, emotional support, and collective identity. Together, these two dimensions were presented as complementary components of an “interdependent well-being” framework. Most professionals (10 out of 12) stated that nature-based interventions foster strong social bonds. One professional highlighted the need to “feel surrounded, to feel like we belong to a community, even if it’s just for the duration of a one-hour activity” (SSI 6), while another described interventions such as NBSP as enabling a “social encounter between inhabitants of the same neighbourhood or building” (SSI 10).

Finally, nature was perceived as a foundational element, “what keeps us alive” (SSI 12), and as a means of connection, helping to “break symbolic barriers” (SSI 3). All but one professional (11 out of 12) described nature as both a vital need for humans and a socially mediating entity, often anchored in familiar and positively valued symbolic meanings. Within the Social Representation Theory framework, this symbolic anchoring positions nature as a universally recognisable and culturally shared reference point that facilitates inclusion, trust, and shared purpose in social interactions. A spiritual dimension to this ontological connection was mentioned by nine professionals across all sectors, and was described as an intrinsic part of the relationship with nature rather than a separate belief system. Nature was described as a superior and autonomous entity that surpasses us, is intrinsically beautiful, and universal across all cultures. One professional stated, “it seems to me that spirituality is completely part of the relationship with nature. We are part of a whole” (SSI 5). This shared vision was seen as favourable to the acceptance of NBSP, which was perceived as a logical extension of this interdependent and holistic perspective. While this shared holistic vision provides a strong conceptual basis for NBSP, professionals also emphasised that its translation into practice is shaped—and often constrained—by the structural inequalities specific to Marseille’s urban environment.

### 3.2. Structural Inequalities in Marseille

This theme comprises two sub-themes: what 10 out of 12 professionals described as a “hostile environment”, referring to disadvantaged neighbourhoods of Marseille, and the unequal distribution of natural spaces. Professionals described Marseille’s urban context as a significant barrier to well-being, pointing to a stark imbalance between concrete and green areas. This is illustrated by comments such as, “In my neighbourhood, there’s concrete everywhere, not even a single tree” (SSI 3). According to professionals, this perception of hostility was fuelled by the degraded state of the built environment, dense housing conditions, traffic congestion, and noise, which together contributed to an atmosphere they perceived as “difficult to live in, noisy, and tiring” (SSI 16). As one professional summarised, “living in Marseille is in itself a factor of stress, and when you add precarity on top of that, it’s really hard to breathe” (SSI 7).

This challenging environment is marked by a starkly unequal distribution of natural resources. This point was highlighted by six professionals, specifically among field actors and institutional representatives from the health and social sectors, and was seen as a social divide. This is highlighted by observations like, “in the chic neighbourhoods there’s greenery, and in the others much less” (SSI 11), framing nature access as a ‘luxury’. Consequently, even major assets like the Calanques National Park, described as a “green lung”, are considered difficult to access for certain populations. Access is limited not only by physical distance but also by a combination of social and symbolic barriers. The theme of symbolic barriers, identified by six professionals from nature institutions, health institutions, and local social actors, refers to situations where residents felt “out of place” in certain spaces or perceived them as “not meant for them.” Past negative experiences sometimes reinforced this sense of exclusion. These structural and symbolic inequalities directly shape the lived realities of the target populations for NBSP, influencing not only their access to nature but also their capacity to participate in community-based health initiatives.

### 3.3. Populations Facing Cumulative Disadvantage

This theme is structured around three sub-themes: the effects of precarity on social isolation and loneliness, specific barriers to participation, and the potential of nature as a resource for social recognition.

Professionals from health institutions, local health actors, and local social actors described the target populations as facing multiple and intersecting forms of disadvantage. They referred to situations of “uprooting” (SSI 1), loss of reference points and social status, often intensified by stigma. One professional described them as a “politically easy target” (SSI 9). This demanding work context, professionals reported, is dominated by a “very short-term vision” (SSI 9), as their efforts are constantly focused on addressing target populations’ primary concerns. Basic physiological needs, such as housing and food, were reported as dominant concerns: “finding housing, food, a roof over their heads” (SSI 6). Several specific obstacles to participation were highlighted, including “language barriers”, “cultural practices” (SSI 9); institutional mistrust: “fear of being caught by the police on the way” (SSI 9); and significant symbolic barriers that confine individuals to their immediate surroundings: “outside of their housing estate they don’t dare to go out, it’s complicated for them… Even their knowledge of their own city…” (SSI 10). According to four professionals from health institutions, nature institutions, and local social actors, this last observation reflects not only practical constraints but also internalised forms of exclusion, illustrating the “symbolic barriers” theme. These descriptions may also reflect a degree of distance between service providers and beneficiaries, underscoring the importance of critically reflecting on professionals’ own positionality to avoid reinforcing an us-versus-them perspective.

Nevertheless, five professionals identified nature as a potential vector for recovery of competence and personal recognition, especially for individuals with a rural background who could reconnect with past agricultural skills. It was seen as a way to restore self-esteem and competencies: “it shows them they haven’t lost their skills” (SSI 1). Some individuals, particularly women, were reported to have explicitly asked professionals for access to natural spaces: “they ask to go out and ask for access to nature” (SSI 4). Addressing these cumulative disadvantages requires strategies that go beyond access, focusing on empowerment and co-construction of solutions with communities.

### 3.4. Pathways Toward Participation and Recognition

This theme comprises two sub-themes: the importance of participatory design and empowerment, and the tensions surrounding the term “prescription.”

The first sub-theme, mentioned by a majority of professionals across all sectors, emphasised the need for a participatory approach, starting with a diagnosis of needs developed with the community itself. They expressed the desire to “start from [people’s] experiences and the lessons they draw from them” (SSI 5), and to build on their internal resources, asking, “How to help the other find their own solutions… starting from their experiences, that’s what’s important” (SSI 5). Some suggested forming a “core group to drive the process” (SSI 12), often composed of both professionals and motivated community members, whose responsibilities could range from co-designing activities to participating in key decision-making steps. Others highlighted the value of “close, hands-on support” (SSI 10), such as accompanying participants during the first sessions, providing regular feedback, or facilitating peer support groups, with the ultimate goal of fostering autonomy. Assigning responsibilities was described as “highly empowering and ego-boosting” (SSI 1).

The second sub-theme, concerning the semantic tension surrounding the term “prescription,” was raised by all actors except local health actors. The critique of the term mainly focused on its association with medical authority and an asymmetrical power dynamic. Some professionals argued, “Prescription sounds like a medical order. That’s not what we want, we want to start from them” (SSI 6), highlighting a resistance to a top-down approach. At the same time, others perceived the prescription as a motivating element: “It provides structure, it’s reassuring for patients, they know it’s not just a walk” (SSI 10). Interestingly, local health actors did not explicitly comment on the term “prescription.” For them, as healthcare professionals, the strength of the programme lies precisely in its capacity to function as supportive care, complementing medical treatment rather than imposing an external mandate. This professional perspective reflects an understanding of prescription not only as a formal directive but as a means to legitimise and integrate the intervention within the healthcare pathway. However, the very terminology used to describe NBSP emerged as a point of contention among professionals.

### 3.5. Barriers and Facilitators to NBSP Implementation

This final theme includes four sub-themes related to territorial constraints, limited institutional resources, identity-based levers, and a shared set of professional values.

Professionals from all sectors identified several structural barriers to the implementation of NBSP. For instance, a lack of nearby green spaces and difficulties accessing natural sites like the Calanques were mentioned by eight professionals. These territorial constraints, often linked to transportation issues, the spatial layout of urban areas, and, in some cases, perceived safety concerns, limit the ability of professionals to fully apply NBSP principles in practice, sometimes forcing adaptations or compromises. Operational constraints also emerged among local actors from health and social sectors, who reported workload saturation: “We’re already overloaded, we can’t do everything” (SSI 8). Furthermore, professionals highlighted the inherent difficulty of mobilising and sustaining involvement over time, noting that for multi-vulnerable individuals, “if you ask people to commit long-term, it’s complicated” (SSI 11).

At the same time, several facilitators were identified. First, the alignment of NBSP principles with existing professional values was perceived as a key lever, noted by most professionals across sectors. This process can be understood as the anchoring of a new social representation (NBSP) within pre-existing professional knowledge, as one professional noted: “We’re not inventing anything new, we’re just putting a framework around what we already do” (SSI 11). This anchoring helped professionals persevere despite external constraints and bridged the semantic and institutional practice levels. Second, nature was unanimously seen as a powerful vehicle for restoring self-esteem: “It helps them regain confidence because it’s not medical, it’s alive” (SSI 1). This motivating factor supports engagement, even when structural barriers exist. Furthermore, NBSP was considered replicable for other populations by professionals from health and nature sectors, as one professional noted: “these people are participating in a pilot project that could later serve other publics” (SSI 7). Finally, a strong sense of social urgency in response to growing loneliness and social isolation reinforced the perceived necessity of the NBSP intervention. Together, these barriers and facilitators illustrate both the structural challenges and the professional commitment that shape the current and future implementation of NBSP, paving the way for a discussion on its scalability and transferability.

Ultimately, results showed that conceptualisations of NBSPs are shaped by two intersecting dimensions: sectoral affiliation (health, social action, urban nature) and hierarchical role (operational vs. decision-making). Operational actors, across all sectors, tended to anchor NBSP in their day-to-day realities, emphasising logistical feasibility, immediate participant needs, and the value of relational proximity. Decision-makers, by contrast, often framed NBSP within strategic and programmatic narratives, focusing on alignment with policy frameworks, institutional mandates, and long-term scalability. Sectoral perspectives further refined these patterns: health professionals tended to interpret NBSP through integrated care and public health paradigms; social action professionals foregrounded empowerment and community development; and urban nature actors emphasised ecological accessibility and environmental justice. These cross-cutting patterns enrich the understanding of how NBSP is anchored across different professional contexts and set the stage for the discussion of its broader implications.

## 4. Discussion

Thematic analysis, interpreted through the lens of Social Representation Theory, highlighted the dynamics through which NBSP is appropriated by mobilising pre-existing knowledge related to nature and health, and social and territorial inequalities. Within this framework, the process of anchoring unfolds along three main axes: (1) a consensual anchoring of NBSP in a holistic vision of health and nature, connected to established health promotion and environmental psychology frameworks; (2) its perceived relevance for addressing Marseille’s pronounced territorial inequalities; and (3) a paradox between participatory ideals and practical barriers to implementation, crystallised around the term “prescription”, which reflects tensions between empowerment and medicalisation.

### 4.1. Anchoring NBSP in a Representation of Nature as a Lever for Biopsychosocial Health Promotion

Receptiveness to the concept of NBSP is closely linked to its anchoring in a widespread social representation of nature as a fundamental pillar of health. This anchoring operates through multiple theoretical traditions: health promotion, which offers a systemic, equity-oriented approach linking social and environmental determinants; environmental psychology, which emphasises nature’s restorative and relational benefits; and the One Health approach, situating these benefits within the broader interdependence of human, animal, and ecosystem health.

In this perspective, nature connection is framed as a determinant of well-being that goes beyond the biomedical model, supporting individual autonomy and social belonging through interaction with natural environments. This understanding aligns with the Ottawa Charter [29] and subsequent health promotion models, which conceptualise health as a complex, intersectoral and multi-level framework involving diverse actors, participation, and empowerment [30]. The benefits of nature align with key theories in environmental psychology, particularly Ulrich’s stress reduction theory and Wohlwill’s characterisation of the qualities of nature-based experiences. Ulrich posits an innate biophilia, whereby nature constitutes an intrinsically soothing and aesthetic environment capable of alleviating the anxiety associated with loneliness [31]. Wohlwill further identifies four experiential qualities of nature: its autonomous character, whose organic growth and transformations inspire wonder; its visual properties, perceived as more ordered and predictable than urban spaces, which foster a sense of safety; its capacity to slow down vigilance mechanisms; and its profound symbolic meanings [32]. For some, the connection between nature and health extends beyond well-being to encompass identity formation, reflecting the principle that nature and the self are closely intertwined across individual life trajectories [33].

The anchoring of NBSP is thus both collective and individual, resonating with deeply personal experiences. In SRT terms, this anchoring not only shapes how nature is understood but also legitimises its use as a lever for health promotion. By acting on both individuals (through emotional relief) and environments (through the creation of opportunities for social interaction), nature addresses loneliness on two levels. Immersion in nature can reduce both emotional and social loneliness by fostering community integration [34]. From this perspective, loneliness is framed as a rupture in the relationship between human well-being and the surrounding social–natural environment. This view resonates with broader contemporary approaches, such as the One Health concept, which emphasises the interdependence of human, animal and ecosystem health [35]. It also foreshadows the perspective of Planetary Health, in which loneliness may be interpreted as a symptom of a fractured relationship between human civilisation and global natural systems [36]. In Marseille, NBSP’s positive reception among professionals reflects not only interest in an innovative intervention, but also a deeper need to repair these human–nature connections. Future research should assess the robustness of this anchoring across sectors and analyse how it is operationalised within institutional constraints.

### 4.2. Territorial Disparities in Marseille as Drivers of Health and Loneliness Inequalities

While the idea of nature as a source of well-being is broadly universal, its application through NBSP is anchored in the specific context of Marseille. NBSP is seen as a targeted response to territorial fractures, which, as shown by the analysis of social determinants of health in the city, directly contribute to residents’ social vulnerability and experiences of loneliness.

The city exhibits marked contrasts between the dense, under-resourced northern districts and the more affluent southern neighbourhoods. This reflects the concept of the social gradient in health, showing how differences in income and living conditions translate into observable disparities in health outcomes [37]. Such a context constitutes a form of environmental injustice, where those most exposed to loneliness due to economic precarity also face limited opportunities for informal social encounters as a result of poor-quality public spaces. From an SRT perspective, these challenges can be understood as symbolic barriers anchored in representations of certain urban areas as inaccessible or socially inappropriate. Past negative experiences may reinforce these perceptions, fostering exclusion and self-stigmatisation, which in turn encourage avoidance behaviours and intensify social isolation. This process creates a second, territorially based determinant of social isolation.

Structural barriers, such as mobility constraints, further exacerbate this dynamic. In Priority Neighbourhoods, limited public transport and the absence of private vehicles for many households reinforce geographic confinement and limit access to larger natural areas [15]. These constraints are perceived not only as practical obstacles but also as disincentives to participation, resulting in avoidance of long or unfamiliar trips, reduced activity participation, and reliance on poor-quality local spaces. From a Social Representation Theory perspective, such constraints anchor NBSP in a representation of Marseille as fragmented, influencing which solutions are deemed legitimate, and favouring proximity-based, culturally adapted interventions. This interpretive lens also shapes which forms of “nature” are perceived as accessible, acceptable, and meaningful for target populations. This territorial dimension of loneliness is inseparable from its psychosocial consequences. As the literature highlights, loneliness is closely associated with stigma and is often experienced through shame and self-deprecation, leading to social avoidance [2,4,5,6]. The relevance of NBSP lies precisely in its potential to address these psychological barriers by reducing fear of judgement, restoring self-esteem, enhancing recognition of individual strengths, and deconstructing internalised obstacles to social connection.

### 4.3. The Tension Between an Empowerment Ideal and a Perceived Top-Down Intervention Model

The implementation of NBSPs among vulnerable populations reveals a central paradox, oscillating between a philosophy grounded in empowerment and a form of pragmatism shaped by structural determinism.

On the one hand, professionals express an intervention ideal strongly aligned with humanistic approaches. Starting from lived experiences and supporting the search for self-defined solutions directly echoes Rogers’ person-centred approach [38]. This ideal corresponds to what is referred to as praxis: an action aimed at recognising the other as the main agent of their own autonomy, particularly in their effort to overcome loneliness [39]. However, this ideal of doing with comes into sharp contrast with an analysis of beneficiaries’ living conditions. To justify the difficulty, or even the impossibility, of fostering participation, frequent reference is made to Maslow’s hierarchy of needs [40]. In their view, it is unrealistic to expect engagement in needs for belonging or self-actualisation, even though these needs are central to alleviating loneliness, when basic physiological and safety needs remain unmet. This reasoning, which attributes vulnerability to external structural factors, aligns with Strauss’s perspective, in which vulnerability is understood as the product of social processes embedded in everyday life [41].

This is where the paradox emerges. While professionals are clearly attuned to the structural determinants of loneliness, some accounts suggest a tendency to reassign responsibility back onto individuals. Within the framework of Social Representation Theory, such categorisation reflects one of the core social functions of representations, namely the classification of individuals and groups in ways that can legitimise existing power relations. In this case, the implicit distinction between participants and non-participants risks reinforcing social boundaries instead of dismantling them. This dynamic risks legitimising exclusion rather than dismantling it, thereby constraining the transformative potential of community-based interventions such as NBSPs. This tension reflects an ambivalent framing of the problem, situated somewhere between structural approaches and individualising models. As suggested by Abric, such a stance may also reflect a representation of the social worker’s role as primarily assisting individuals in difficulty, rather than engaging in complex efforts to dismantle mechanisms of social exclusion [42]. This ambivalence underscores the need for a paradigm shift in health promotion, favouring community-based and systemic action, such as that proposed by NBSP.

The paradox between participatory ideals and pragmatic constraints is clearly illustrated by the semantic debate surrounding the term “prescription”. The term is far from neutral. It crystallises tensions between two models of intervention and reveals competing professional cultures. In the nature and social sectors, it is anchored in a representation of medical authority that is hierarchical and potentially exclusionary, often perceived as paternalistic and incompatible with empowerment logics. In the health sector, by contrast, “prescription” fits more easily into care pathways, conveying legitimacy and structured support. In SRT terms, these contrasting interpretations reflect two anchoring processes: one embedding the term in integrated care pathway representations, the other in representations of medical authority. In line with previous work in health promotion, practitioners have suggested replacing the term “prescription” with alternative expressions such as “referral” or “well-being pathway,” which could better align with participatory and empowerment-oriented approaches by reducing the imprint of medical authority. Considering such terminology shifts could help broaden NBSP’s legitimacy and acceptability across both professional sectors and community stakeholders.

These divergences show how terminological choices can reproduce sectoral boundaries and power relations. They also illustrate how language can be used strategically to align NBSP with participatory health promotion principles. Ultimately, this tension risks shifting NBSP from a collective, structural intervention towards an individualising model. Yet loneliness is linked not only to individual experiences but also to social networks, civic participation, and social recognition. By mobilising nature as a non-stigmatising third space that fosters collective narratives and strengthens cohesion, NBSP can reduce emotional loneliness, restore self-worth, and reactivate local social dynamics.

Despite the paradoxes observed, NBSP retains the potential to repair social bonds, transforming loneliness into an experience that can be shared, tolerated, and even used as a foundation for mental well-being.

## 5. Limits

This study presents several contextual, methodological, and epistemological limitations. First, the specific context of Marseille, marked by significant territorial disparities, a particular urban geography, and high levels of social precarity, constitutes both a source of analytical richness and a limiting factor for the transferability of the findings to other settings.

From a methodological perspective, the sample size, although aligned with qualitative standards in terms of thematic saturation [26], does not allow for generalisation of the results, particularly regarding the relationship between professional affiliation and social representations and the specific context of the study, conducted in Marseille. Furthermore, the absence of interviewee feedback (member checking), although justified by the intention to preserve the spontaneity of responses, limits the external validation of the interpretations.

A further limitation is the absence of direct perspectives from beneficiaries in this phase of the study. However, the participatory diagnostic phase of the RECETAS project involved co-constructing a menu of NBSP activities through participatory methods, thereby including beneficiaries. This process allowed for a more refined understanding of the needs of those directly concerned, while the present study specifically focused on understanding how NBSP is integrated into professional practices.

Regarding the analysis, although conducted with rigour, it inevitably involves a degree of subjectivity inherent in qualitative approaches. The complementary use of textual analysis software could have further enriched the interpretation. Likewise, while the decision to integrate free associations into the overall thematic analysis is consistent with an interpretative stance, some readers might question the absence of a distinct structural or prototypical analysis, which could have shed additional light on the underlying socio-cognitive dimensions.

### Practical Implications for NBSP Implementation

This study led to the formulation of a set of operational recommendations that subsequently guided the implementation of NBSP in Marseille. Rooted in both the specific territorial context and the principles of health promotion, these actions aimed to strengthen the relevance, effectiveness, and equity of NBSP as a strategy to address loneliness.

To enhance accessibility, NBSP was primarily implemented in priority neighbourhoods, in partnership with locally embedded professionals. A set of progressive and adaptable activities, such as nature walks and gardening workshops, was co-designed with professionals and the target populations during the second and third phases of the co-creation process of RECETAS. These included a participatory diagnostic phase and the collective definition of a panel of NBSP activities. Five focus groups supported this process, which aimed not only to foster empowerment by valuing individuals’ own resources but also to build a shared culture around care, social connection, and nature. Professional reflexivity was also supported through interprofessional exchange sessions designed to foster a common culture. Finally, a mixed-methods evaluation was implemented to assess lived experience, changes in perceived loneliness, well-being and quality of life outcomes. By integrating environmental, social, cultural, and psychological dimensions, NBSP emerges as a systemic response to the multidimensional determinants of loneliness. This study highlights that this approach is positively received by professionals due to its grounding in a holistic view of health and in shared representations of nature as a source of well-being and social connection. In relation to the semantic discussions raised earlier, at this stage of the project, and in coherence with the other pilot teams involved in RECETAS, no change in terminology has been implemented locally. The decision to retain or adapt the term “prescription” will be revisited collectively at the end of the project, once all pilot sites have gathered empirical evidence on its acceptability and impact. This staged approach allows for cross-context learning while maintaining a consistent vocabulary during the initial implementation phase.

## 6. Conclusions

In the Marseille context, marked by significant territorial inequalities, NBSP is perceived as an innovative lever for disrupting mechanisms of social exclusion. The uniqueness of this territory lies in the coexistence of diverse natural environments, offering a distinctive potential to create spaces for social encounters where nature can function as a relational third place, safe and non-stigmatising. It is precisely this collective dimension that NBSP aims to strengthen by facilitating the emergence of new shared narratives and reinforcing psychosocial cohesion at the local level. Its anticipated effects include a reduction in emotional loneliness (by alleviating the feeling of facing difficulties alone), subjective enhancement (through a renewed sense of belonging and social usefulness), and reconnection with others through the reactivation of localised social dynamics (renewed contacts, integration into other activities). Thus, despite the paradoxes identified and supported by other works that have studied the representation of nature in Marseille and shown both the richness of its peripheral natural spaces and a fragmented urban nature, ref. [43] NBSP may act as a genuine mechanism for repairing social bonds, contributing to a transformation of loneliness into a shareable, more tolerable experience, and even a potential foundation for the restoration of mental well-being. However, this dynamic encounters structural tensions. Although professionals advocate for participatory ideals, they are confronted with the material and psychological constraints of the target populations, which may lead to more top-down forms of intervention than initially envisioned. These findings invite us to consider NBSP not merely as a sector-specific innovation but as an opportunity to renew public health paradigms.

Firstly, it suggests the need to explicitly frame the reduction in emotional loneliness and the strengthening of social belonging as central objectives. To achieve its full potential, NBSP must move beyond the boundaries of individual interventions and be embedded within a collective and contextualised strategy, supported by ambitious public policies and training programmes that promote professional reflexivity and active beneficiary involvement. Secondly, NBSP, as a new social object and intervention in France, derives its meaning from a deep interdependence between health, natural spaces, and social relations. The recognition of this interdependence implies an integration of human and ecosystem health, a concept known as Planetary Health [36]. This holistic and integrative representation of health, through which the interviewed professionals understand NBSP, also reflects a shift towards an ecological model of public health [44]. This new ecological public health transcends individualistic preventive and curative approaches and, much like the field of health promotion, calls for a consideration of social determinants of health at the macro level and an expanded responsibility for health across disciplines beyond traditional healthcare boundaries.

A further significant finding is the profoundly relational character of NBSP, where nature is conceptualised as a resource for developing social bonds. This characteristic suggests reframing ecology itself (and by extension, the ecological public health model previously cited) in a resolutely relational orientation, thinking of ecology as the possibility of forging connections between humans, and between humans and natural spaces [45]. This notion is supported by previous research on the social representations of nature, which has highlighted the importance of introducing relational values (between people and between people and nature) to develop systemic strategies around nature and human well-being [46].

## Figures and Tables

**Table 1:** Distribution of the sample according to professional affiliation.

Interview	Profession	Organisation	NBSP Domain
1	Educator (o)	Social support drop-in centre	S
2	Coordinator (o)	Local non-profit association	S
3	General practitioner (o)	Primary healthcare unit	H
4	Manager (dm)	Regional public health agency	H
5	Head of service (o)	Shelter and social reintegration centre	S
6	Project officer (dm)	Municipal urban agriculture initiative	UN
7	Project officer (o)	Municipal urban agriculture initiative	UN
8	Coordinator (dm)	Public health and urban health programme	H
9	General practitioner (o)	Hospital-based healthcare unit	H
10	Project officer (dm)	Regional public health agency	UN
11	Coordinator (dm)	Municipal environmental department	H
12	Volunteer, Association President (o)	Community gardening association	UN

NBSP domain: H = Health; S = Social action; UN = Urban nature. Role: (dm) = Decision-making; (o) = Operational. Participants were selected to ensure coverage of all three domains and representation of both decision-making and operational roles.

**Table 2:** Thematic structure of the analysis: key themes and sub-themes regarding the implementation of NBSP.

Theme		Sub-Themes	
I.	A holistic conception of health, community, and nature	1	Health as a multidimensional and interconnected phenomenon
		2	The need for social belonging as a core human experience
		3	Perceived continuity between humans and nature
		4	Nature-based activities as catalysts for social connection
II.	Structural inequalities in Marseille	5	Urban living conditions as a barrier to well-being
		6	Reproduction of social inequalities through unequal access to nature
III.	Populations facing cumulative disadvantage	7	A focus on basic needs limiting long-term engagement
		8	Expressions of psychosocial distress among target groups
IV.	Pathways toward participation and recognition	9	Conditions for meaningful public participation
		10	Recognition of local knowledge and lived experience
V.	Barriers and facilitators to NBSP implementation	11	Difficulties in engaging structurally isolated populations
		12	Institutional constraints and top-down dynamics
		13	Enablers of NBSP implementation

## Data Availability

The data presented in this study are available on request from the corresponding author due to privacy and ethical considerations.
